# Clinical impact of the prognostic nutritional index at listing and immediately before surgery in lung transplantation

**DOI:** 10.1007/s00595-026-03421-x

**Published:** 2026-07-24

**Authors:** Shinji Yuhara, Gouji Toyokawa, Junichi Takada, Miho Yamaguchi, Yue Cong, Takaki Akamine, Masahiro Yanagiya, Takahiro Karasaki, Mitsuaki Kawashima, Chihiro Konoeda, Yan Luo, Masaaki Sato

**Affiliations:** 1 Department of Thoracic Surgery, The University of Tokyo Graduate School of Medicine, 7-3-1 Hongo, Bunkyo-ku, Tokyo, 113-8655 Japan; 2 Department of Surgery and Science, Graduate School of Medical Sciences, Kyushu University, 3-1-1, Maidashi, Higashi-ku, Fukuoka, 812-0054 Japan; 3 Department of Respiratory Medicine, The University of Tokyo Graduate School of Medicine, 7-3-1 Hongo, Bunkyo-ku, Tokyo, 113-8655 Japan; 4 Department of Next-Generation Organ Transplantation, The University of Tokyo Graduate School of Medicine, 7-3-1 Hongo, Bunkyo-ku, Tokyo, 113-8655 Japan

**Keywords:** Lung transplantation, Prognostic nutritional index, Nutrition, Survival, Waiting list

## Abstract

**Purpose:**

The prognostic nutritional index (PNI), which is calculated from the serum albumin concentrations and lymphocyte counts, predicts the outcomes in lung transplantation (LT). This study aimed to investigate the optimal timing for assessing PNI before LT.

**Methods:**

We analyzed 140 brain-dead LT recipients (2015–2023) in this study. The PNI was calculated at the time of transplant listing and immediately before LT. Using a cutoff value of 45, the patients were classified into high/low PNI groups and compared for overall survival. Changes in the PNI trajectories were analyzed.

**Results:**

Multivariable analyses showed that a low PNI independently predicted mortality at listing (hazard ratio [HR], 2.82; 95% confidence interval [CI], 1.44–5.53; *P* = 0.003) and before LT (HR, 2.44; 95% CI, 1.25–4.77; *P* = 0.009). Patients with a persistently low PNI had a worse survival than those with a persistently high PNI (HR, 3.58; 95% CI, 1.62–7.91; *P* = 0.002), whereas an improvement in the PNI after listing was not significantly associated with survival.

**Conclusion:**

PNI at both time points significantly predicted post-transplant outcomes. A trajectory analysis highlights the importance of nutritional assessment before listing, although larger studies are required to clarify the effects of nutritional changes after listing.

**Supplementary Information:**

The online version contains supplementary material available at https://doi.org/10.1007/s00595-026-03421-x.

## Introduction

The preoperative nutritional status has been recognized as an important predictor of postoperative outcomes in various fields, including surgical oncology and lung transplantation (LT) [1–7]. The prognostic nutritional index (PNI), originally developed by Onodera et al. in 1984 to assess the risk of postoperative complications in malnourished patients undergoing gastrointestinal surgery, is calculated using serum albumin concentrations and peripheral lymphocyte counts to provide a simple assessment of the nutritional and immunological status [8]. The PNI has demonstrated prognostic value across various malignancies [1, 2] and it has been applied to solid organ transplantation. In the field of LT, PNI has been shown to be a prognostic factor for post-transplant outcomes [9–13].

In regions with prolonged waiting periods, such as Japan, where candidates wait a median of 708 days for brain-dead donor LT [14], nutritional deterioration during the waiting period may negatively affect the postoperative outcomes, including survival. This possibility suggests the importance of nutritional intervention. While some studies have evaluated nutritional status at the time of transplant listing [10, 11], others have focused on the preoperative status, often without a clear description of the exact timing of assessment [9, 13]. Therefore, the optimal timing for assessing the nutritional status to predict post-transplant survival remains unclear. Furthermore, the effects of changes in nutritional status over time on postoperative outcomes remain unclear.

The present study aimed to evaluate the prognostic value of the PNI measured at listing and immediately before LT, to evaluate whether changes in the PNI during the waiting period affect the post-transplant outcomes, and thereby to examine the potential benefit of nutritional management during the waiting period.

## Methods

### Study design and patient selection

The study was approved by the Institutional Review Board (IRB# 2406). We conducted a retrospective cohort study of brain-dead LTs performed at our institution between January 2015 and December 2023. From an initial cohort of 150 consecutive brain-dead lung transplant recipients, we excluded eight with missing data required for calculating the PNI and two who underwent re-transplantation. Therefore, the final analysis included 140 patients in total.

### Data collection and PNI calculation

The clinical data were extracted from our transplant database. Age, sex, and primary diagnosis were recorded at listing, while data on the transplant type (single or bilateral) and waiting time were collected at transplantation. The serum albumin concentrations, total lymphocyte count, and body mass index were measured at two time points: at listing and immediately before LT (within 24 h). The PNI was calculated using the following formula: PNI = 10 × serum albumin (g/dL) + 0.005 × total lymphocyte count (/mm³), which is equivalent to PNI = serum albumin (g/L) + 5 × lymphocyte count (× 10⁹/L). Intraoperative variables, including operative time, blood loss, ischemic time, and use of extracorporeal membrane oxygenation, were also collected.

The short-term outcomes included intensive care unit stay, hospital stay, ventilation duration, and perioperative mortality. Failure to wean from mechanical ventilation was defined as the inability to achieve liberation from mechanical ventilation before in-hospital death or the first hospital discharge. The duration of ventilation was set as the survival period or the length of hospital stay. The long-term follow-up outcome was overall survival (OS), defined as the time from transplantation to death from any cause. OS data were collected until January 31, 2026. Perioperative management followed our institutional standard protocol throughout the study period, with no major changes in the surgical technique or immunosuppression.

### Statistical analysis

Statistical analyses were conducted in two parts: evaluation of the prognostic value of the PNI measured at listing and immediately before LT, and assessment of the association between changes in the PNI during the waiting period and post-transplant outcomes.

### Prognostic value of the PNI at listing and immediately before LT

We evaluated the prognostic value of the PNI measured at listing and immediately before LT. At each time point, the PNI was dichotomized using a cutoff value of 45, with patients classified as having a low (PNI < 45) or high (PNI ≥ 45) nutritional status. This cutoff was selected based on several considerations. Previous LT studies have reported PNI thresholds ranging from approximately 41 to 48 [9–13], indicating heterogeneity without an established consensus. Among these, receiver operating characteristic (ROC) curve analyses identified cutoffs of 45.8 for waitlist mortality [11] and 46.35 for post-transplant survival [12], with both cutoffs approximately 45. We adopted 45 as a clinically pragmatic threshold for the present analysis because of the limited consensus in the transplantation field and the extensive evidence linking the PNI to long-term postoperative survival in surgical oncology, where a cutoff of 45 has been widely used and validated in large-scale studies [8, 15–18]. An ROC curve analysis was also performed to assess the discriminative ability of PNI at each time point for overall mortality, and the area under the curve (AUC) with 95% confidence interval (CI) was calculated.

At each time point, OS was the primary endpoint, and short-term postoperative outcomes were the secondary endpoints. Survival curves were generated using the Kaplan–Meier method with differences assessed by the log-rank test. To assess the robustness of the PNI–survival association across different adjustment sets, we fitted three multivariable Cox proportional hazards models for the PNI at listing and immediately before LT separately. Model 1 was adjusted for age and sex, serving as a descriptive baseline. Model 2 was additionally adjusted for primary disease, the most clinically established prognostic factor in lung transplantation. Primary disease was categorized as interstitial lung disease (ILD, comprising idiopathic interstitial pneumonias and connective tissue disease-ILD [CTD-ILD]) versus non-ILD. Model 3 was additionally adjusted for transplant type (bilateral versus single). Model 2 was adopted as the primary analysis to balance causal inference with the modest sample size, and Models 1 and 3 were performed as sensitivity analyses. Secondary outcomes were analyzed at each time point, using the Mann-Whitney U test or Kruskal-Wallis test for continuous variables, and the chi-square test or Fisher’s exact test for categorical variables, as appropriate.

### Effect of the PNI trajectory on survival

Patients were classified into four trajectory patterns based on serial PNI assessments at listing and immediately before LT using the same cutoff value of 45: low-low, low-high, high-low, and high. We focused on the long-term outcomes because of the exploratory nature of our analysis.

Survival curves for the four patterns were first visualized using the Kaplan–Meier method and compared using the log-rank test. To perform pairwise comparisons between the four groups, we used univariable Cox regression with Bonferroni correction for multiple comparisons (significance threshold: *P* < 0.008 for six comparisons). We then estimated the hazard ratio (HR) for each pairwise comparison using a multivariable Cox proportional hazards model adjusted for age, sex, and primary disease, and applied the same Bonferroni correction.

All statistical analyses were performed using R version 4.3.0 (R Foundation for Statistical Computing, Vienna, Austria). The proportional hazards assumption for all Cox models was assessed by visual inspection of the Kaplan–Meier curves and confirmed using the Schoenfeld residual test. In the sensitivity analysis, the PNI was modeled as a continuous variable using the same multivariable Cox models. Statistical significance was set at P value < 0.05.

## Results

### Patients characteristics

**Fig. 1:**
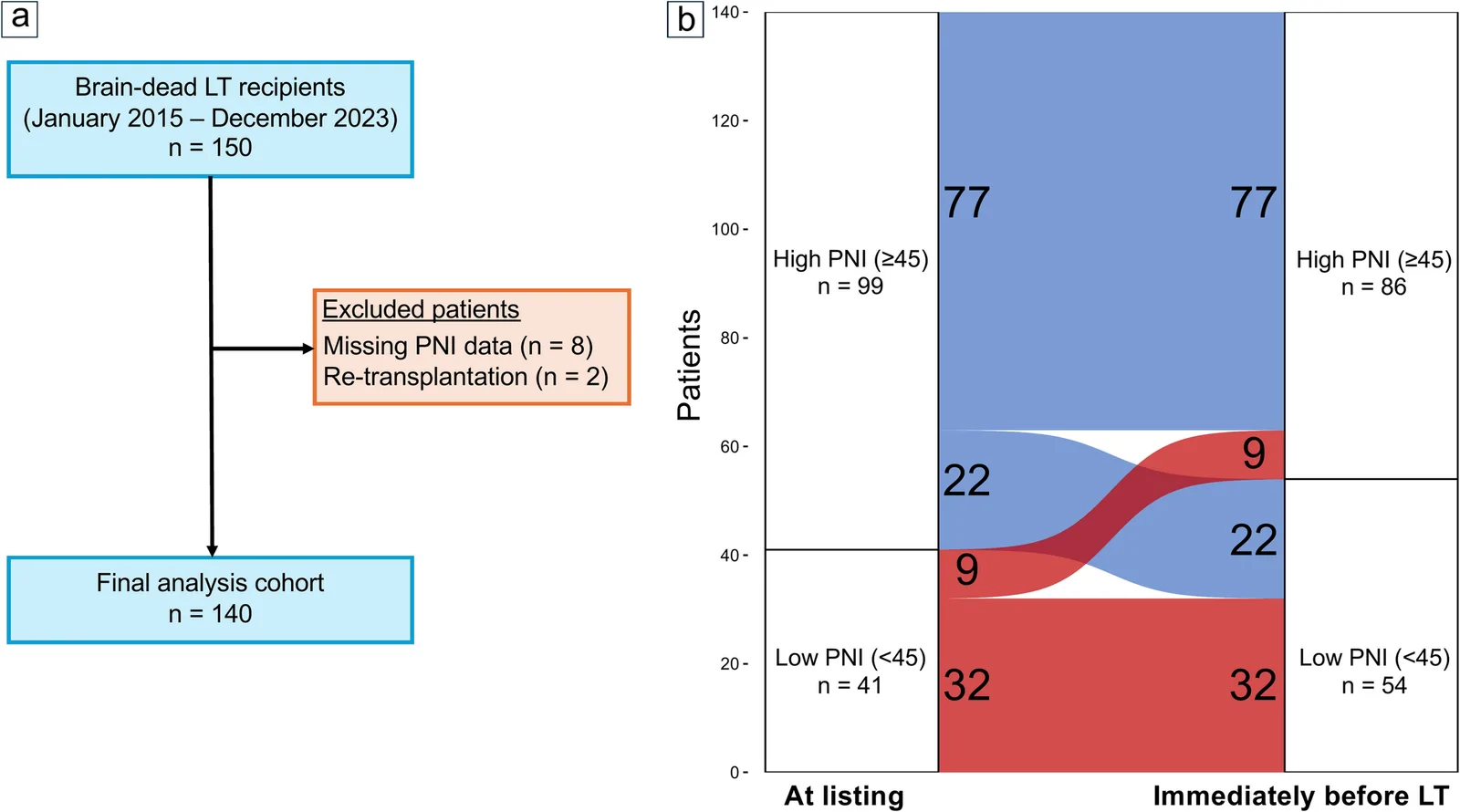
**(a)** Flow diagram of the patients’ selection. (**b**) Sankey diagram showing the trajectory of the prognostic nutritional index from listing to lung transplantation. The patients were stratified into the low PNI (< 45) and high PNI (≥ 45) groups at each time point

The study cohort comprised 140 lung transplant recipients (Fig. 1a). The patients’ baseline characteristics are presented in Table 1. The median age was 47 years, with first and third interquartile ranges of 33 and 55 years, respectively, and the proportion of male patients was 56.4%. Bilateral LT was performed in 79 (56.4%) patients. The most common indications were idiopathic interstitial pneumonia (41.4%), pulmonary hypertension (15.7%), and CTD-ILD (12.1%). The median follow-up period was 45 months (range, 1–126 months).

**Table 1:** Patient characteristics stratified by PNI at listing and before LT

	All cases (*n* = 140)	PNI at listing			PNI before LT		
		PNI low (*n* = 41)	PNI high (*n* = 99)	*P* value	PNI low (*n* = 54)	PNI high (*n* = 86)	*P* value
Age, years (IQR)	47.0 (33.0, 55,0)	49.0 (42.0, 58.0)	46.0 (30.0, 55.0)	0.03	50.0 (40.3, 56.8)	45.5 (28.0, 54.8)	0.06
Male, n (%)	79 (56.4)	27 (65.9)	52 (52.5)	0.21	29 (53.7)	50 (58.1)	0.73
Bilateral LT, n (%)	79 (56.4)	19 (46.3)	60 (60.6)	0.17	31 (57.4)	48 (55.8)	0.99
BMI at listing (IQR)	19.9 (16.8, 23.7)	21.2 (16.1, 26.2)	19.6 (17.0, 23.6)	0.68	19.4 (16.4, 23.5)	20.5 (17.1, 23.7)	0.58
BMI before LT (IQR)	18.8 (16.1, 22.9)	19.0 (16.0, 24.3)	18.7 (16.2, 22.2)	0.68	18.0 (15.7, 21.7)	19.7 (16.4, 23.7)	0.15
PNI at listing (IQR)	47.7 (44.1, 51.7)	41.84 (39.65, 43.56)	50.35 (47.12, 53.09)	-	43.65 (41.58, 46.05)	50.40 (46.97, 53.45)	-
PNI before LT (IQR)	46.8 (42.5, 50.9)	41.76 (39.22, 43.67)	48.34 (45.43, 51.35)	-	41.14 (39.25, 43.32)	50.07 (47.48, 51.90)	-
Waiting period, months (IQR)	26.0 (13.0, 33.0)	26.0 (13.0, 31.0)	26.0 (13.5, 33.0)	0.82	26.0 (11.5, 31.8)	26.0 (15.0, 34.0)	0.44
Intraoperative variables							
Operative time, min (IQR)	515 (373, 644)	522 (368, 692)	513 (379, 634)	0.78	561 (375, 742)	509 (371, 572)	0.10
Blood loss, mL (IQR)	1695 (825, 3235)	1555 (780, 4750)	1820 (850, 3148)	0.92	1895 (860, 5620)	1588 (788, 2825)	0.16
Ischemic time, min (IQR)	467 (411, 566)	431 (379, 572)	482 (421, 563)	0.20	470 (396, 574)	462 (417, 561)	0.88
ECMO use, n (%)	98 (70.0)	27 (65.9)	71 (71.7)	0.63	37 (68.5)	61 (70.9)	0.91
Primary disease, n (%)				0.11			0.08
IIPs	58 (41.4)	19 (46.3)	39 (39.4)		25 (46.3)	33 (38.4)	
CTD-ILD	17 (12.1)	10 (24.4)	7 (7.1)		10 (18.5)	7 (8.1)	
PH	22 (15.7)	3 (7.3)	19 (19.2)		4 (7.4)	18 (20.9)	
Bronchiectasis	10 (7.1)	3 (7.3)	7 (7.1)		5 (9.3)	5 (5.8)	
GVHD	8 (5.7)	2 (4.9)	6 (6.1)		3 (5.6)	5 (5.8)	
COPD	7 (5.0)	1 (2.4)	6 (6.1)		1 (1.9)	6 (7.0)	
BO	4 (2.9)	0 (0.0)	4 (4.0)		0 (0.0)	4 (4.7)	
Others	14 (10.0)	3 (7.3)	11 (11.1)		6 (11.1)	8 (9.3)	

Abbreviations: IQR, interquartile range; LT, lung transplantation; BMI, body mass index; PNI, prognostic nutritional index; ECMO, extracorporeal membrane oxygenation; IIPs, idiopathic interstitial pneumonias; PH, pulmonary hypertension; CTD-ILD, connective tissue disease-interstitial lung disease; GVHD, graft-versus-host disease; COPD, chronic obstructive pulmonary disease; BO, bronchiolitis obliterans. “Before LT” indicates immediately before lung transplantation (within 24 h). Values are median (IQR) or n (%). PNI cutoffs: 45. P-values calculated using Mann-Whitney U test for continuous variables, and chi-square test or Fisher’s exact test for categorical variables, as appropriate

When stratified by the PNI at listing using a cutoff of 45, then 41 patients were classified as having a low PNI and 99 as having a high PNI. Using the same cutoff immediately before LT, 54 patients were classified as having a low PNI and 86 as having a high PNI. The trajectory of the PNI status between these two time points is shown in Fig. 1b. The patients’ characteristics stratified by the PNI are shown in Table 1. No significant difference in the median waiting time was observed at listing (*P* = 0.82) or immediately before LT (*P* = 0.44) between the low and high PNI groups. At listing, patients in the low PNI group were significantly older than those in the high PNI group (*P* = 0.03). No significant differences in intraoperative variables were observed between the PNI groups at either time point. The distribution of primary diseases differed between the groups. In the low PNI group, pulmonary hypertension was less prevalent (7.3% vs. 19.2%), but CTD-ILD was more common (24.4% vs. 7.1%) than those in the high PNI group. Immediately before LT, patients in the low PNI group tended to be older than those in the high PNI group (*P* = 0.06). The distribution of primary diseases also differed between the groups. In the low PNI group, pulmonary hypertension was less prevalent (7.4% vs. 20.9%) but CTD-ILD was more common (18.5% vs. 8.1%) than those in the high PNI group.

### Prognostic value of the PNI at listing and immediately before LT

**Fig. 2:**
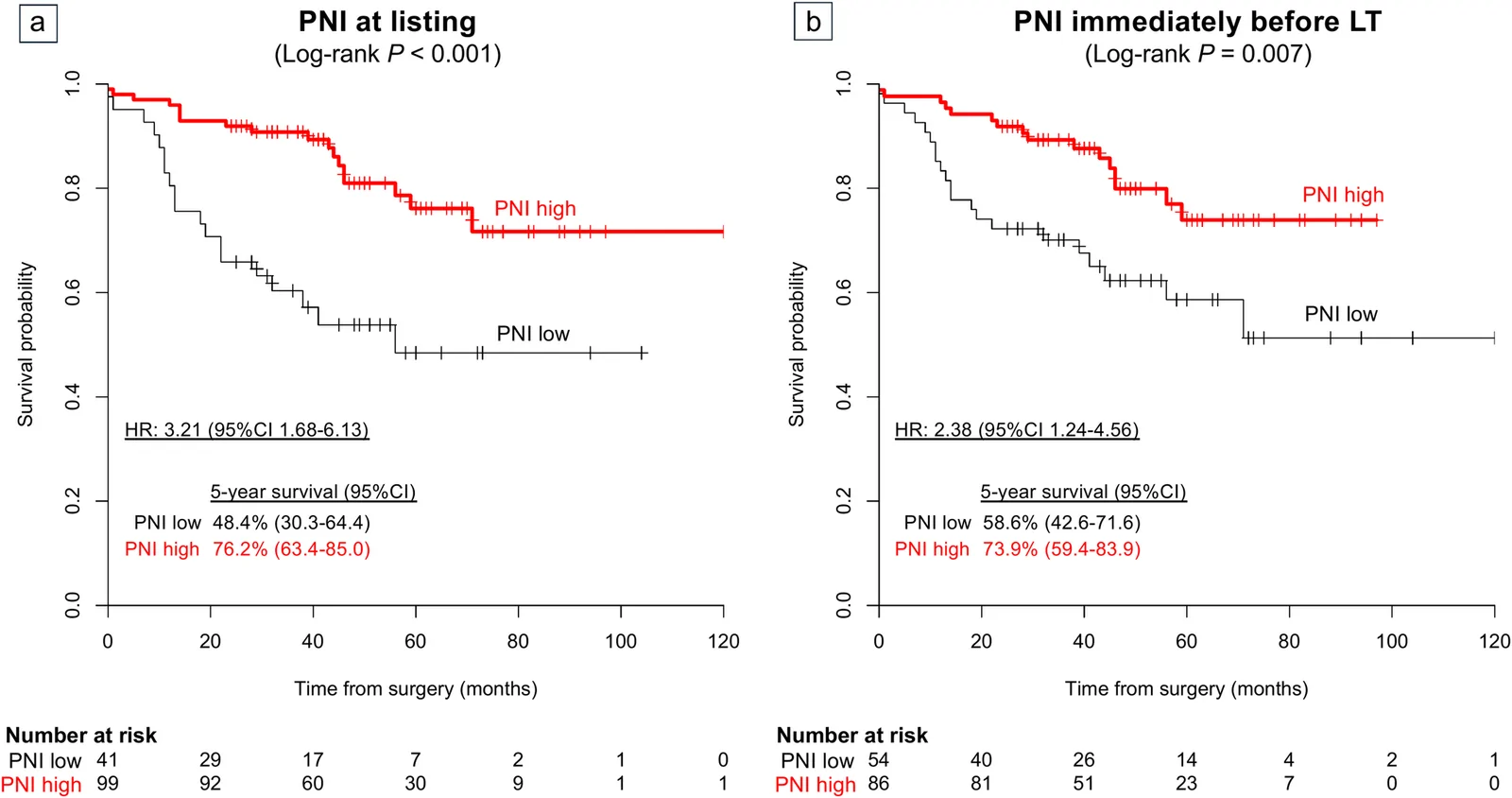
Overall survival curves stratified by the prognostic nutritional index (**a**) at listing and (**b**) immediately before lung transplantation

The AUC for PNI at listing was 0.612 (95% CI, 0.495–0.729) (Supplementary Fig. 1a) and that for PNI before LT was 0.641 (95% CI, 0.534–0.748) (Supplementary Fig. 1b). The log-rank test showed a significantly worse OS in patients in the low PNI group at listing than in those in the high PNI group (*P* < 0.001) (Fig. 2a). In the univariable Cox regression analysis, a low PNI was also associated with a poorer OS (HR, 3.21; 95% CI, 1.68–6.13; *P* < 0.001). The 5-year OS probabilities were 48.4% in the low PNI group (95% CI, 30.3%–64.4%) and 76.2% in the high PNI group (95% CI, 63.4%–85.0%). Similarly, immediately before LT, patients in the low PNI group had a significantly worse OS than those in the high PNI group (log-rank *P* = 0.007), with a corresponding HR of 2.38 (95% CI, 1.24–4.56; *P* = 0.009; Fig. 2b). The 5-year OS probabilities were 58.6% in the low PNI group (95% CI, 42.6%–71.6%) and 73.9% in the high PNI group (95% CI, 59.4%–83.9%). In the primary multivariable Cox regression analysis (Model 2, adjusted for age, sex, and primary disease), a low PNI remained an independent predictor of mortality at listing (HR, 2.82; 95% CI, 1.44–5.53; *P* = 0.003) and immediately before LT (HR, 2.44; 95% CI, 1.25–4.77; *P* = 0.009) (Table 2). The estimates for a low PNI were consistent across Models 1 (age, sex), 2, and 3 (additionally adjusted for transplant type), supporting the robustness of the association (Supplementary Table 1). Visual inspection of the Kaplan–Meier curves showed approximately parallel patterns between groups, with no crossing observed, and the Schoenfeld residual test confirmed that the proportional hazards assumption was satisfied for both Cox models (global *p* = 0.328 for the PNI at listing model and global *p* = 0.294 for the PNI immediately before LT model). In a sensitivity analysis treating the PNI as a continuous variable, a lower PNI remained independently associated with worse survival at listing (HR, 1.06 per 1-unit decrease; 95% CI, 1.01–1.12; *P* = 0.04) and immediately before LT (HR, 1.08; 95% CI, 1.02–1.15; *P* = 0.01), consistent with the primary analysis (Supplementary Table 2).

**Table 2:** Multivariable Cox regression analysis for overall survival

	PNI at listing			PNI before LT		
	HR	95% CI	*P* value	HR	95% CI	*P* value
Age, years	0.99	0.96–1.01	0.29	0.99	0.96–1.01	0.35
Low PNI	2.82	1.44–5.53	0.003	2.44	1.25–4.77	0.009
Male	2.31	1.08–4.94	0.03	2.67	1.24–5.73	0.01
ILD	2.25	1.03–4.91	0.04	2.33	1.05–5.16	0.04

Abbreviations: HR, hazard ratio; CI, confidence interval; LT, lung transplantation; PNI, prognostic nutritional index; ILD, interstitial lung disease. “Before LT” indicates immediately before lung transplantation (within 24 h). Primary disease was categorized as ILD (comprising idiopathic interstitial pneumonias and connective tissue disease-associated interstitial lung disease) versus non-ILD. The multivariable Cox regression model was adjusted for age, sex, and primary disease (ILD vs. non-ILD). Total number of death events: 37

### Postoperative short-term outcomes

The short-term outcomes stratified by the PNI at listing and immediately before LT are shown in Table 3. No significant differences were observed in the short-term outcomes between the groups. However, there tended to be a higher proportion of patients who could not be weaned from mechanical ventilation at discharge or death in the low PNI group than in the high PNI group (*P* = 0.08 at listing and *P* = 0.07 immediately before LT).

**Table 3:** Short-term postoperative outcomes stratified by PNI at listing and before LT

	PNI at listing			PNI before LT		
	PNI low (*n* = 41)	PNI high (*n* = 99)	*P* value	PNI low (*n* = 54)	PNI high (*n* = 86)	*P* value
ICU stay, days (IQR)	12.0 (7.0, 26.0)	13.0 (7.0, 22.0)	0.88	14.5 (7.3, 26.8)	12.0 (7.0, 21.0)	0.38
Hospital stay, days (IQR)	50.0 (36.0, 102.0)	46.0 (34.5, 60.5)	0.26	47.5 (38.3, 105.8)	45.0 (34.3, 57.0)	0.11
ICU mortality, n (%)	2 (4.9)	1 (1.0)	0.43	2 (3.7)	1 (1.2)	0.68
In-hospital mortality, n (%)	3 (7.3)	3 (3.0)	0.50	4 (7.4)	2 (2.3)	0.31
Failure to wean from mechanical ventilation, n (%)	5 (12.2)	3 (3.0)	0.08	6 (11.1)	2 (2.3)	0.07
Ventilation period, days (IQR)	5.0 (3.0, 34.0)	5.0 (3.0, 19.5)	0.95	6.0 (3.0, 26.8)	5.0 (3.0, 19.8)	0.35

Abbreviations: ICU, intensive care unit; LT, lung transplantation; PNI, prognostic nutritional index. “Before LT” indicates immediately before lung transplantation (within 24 h). Values are median (IQR) or n (%). P-values calculated using Mann-Whitney U test for continuous variables and chi-square test for categorical variables

### Effect of the PNI trajectory on survival

**Fig. 3:**
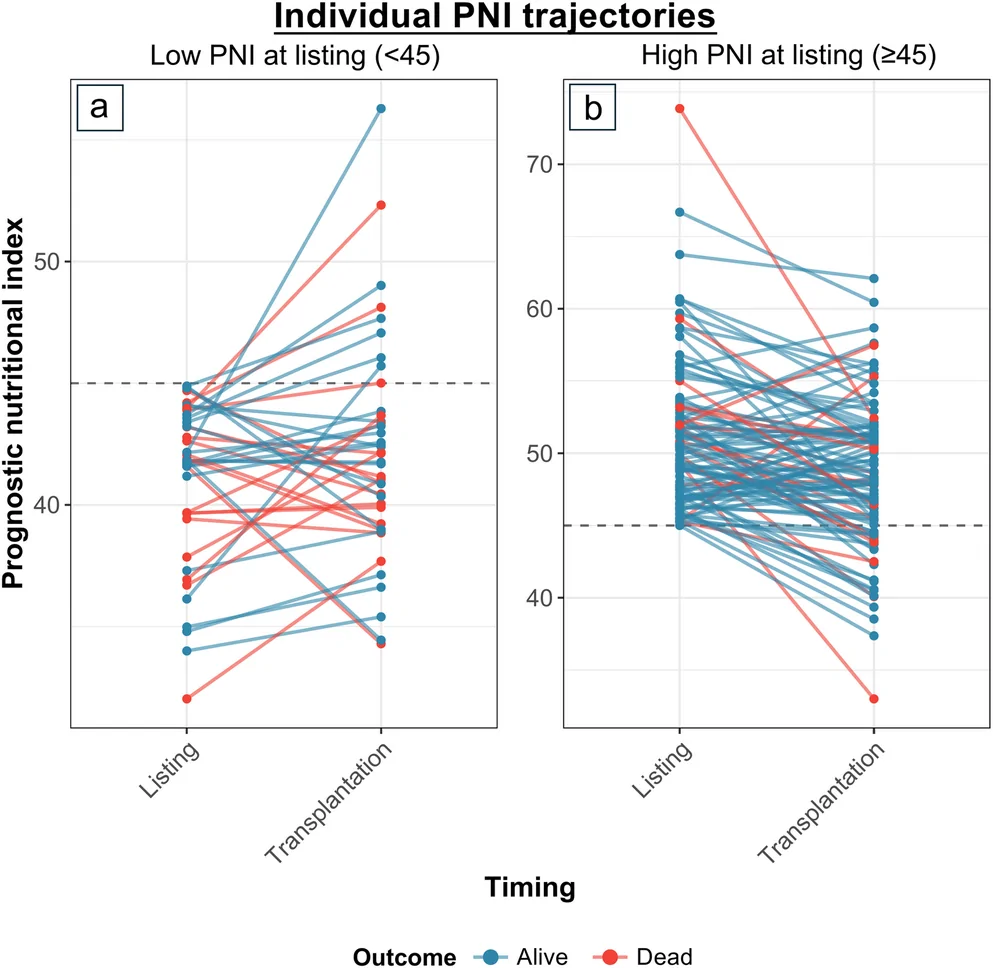
Spaghetti plots of the prognostic nutritional index trajectories from listing to lung transplantation. Individual patient’s trajectories of the prognostic nutritional index (PNI) from listing to lung transplantation, stratified by the PNI at listing. The horizontal line indicates the cutoff value of 45. (a) Low PNI group (< 45). (b) High PNI group (≥ 45)

In all patients, the median change in the PNI value was − 1.3 (interquartile range, − 4.6 to + 1.7), and the median percentage change was − 2.6% (interquartile range, − 8.6% to + 4.2%) between the two time points. The individual PNI trajectories categorized by the PNI values at listing are shown in Fig. 3.

A total of 32, 9, 22, and 77 patients were classified into the low-low, low-high, high-low, and high PNI trajectory groups, respectively. The baseline characteristics of each group are shown in Table 4. No significant differences in baseline characteristics were observed among the groups, except for the PNI.

**Table 4:** Patient characteristics stratified by PNI trajectory groups

	Low-Low (*n* = 32)	Low-High (*n* = 9)	High-Low (*n* = 22)	High-High (*n* = 77)	*P* value
Age, years (IQR)	50.0 (44.5, 58.5)	43.0 (40.0, 58.0)	48.0 (31.3, 55.0)	46.0 (28.0, 54.0)	0.13
Male, n (%)	20 (62.5)	7 (77.8)	9 (40.9)	43 (55.8)	0.23
Bilateral LT, n (%)	16 (50.0)	3 (33.3)	15 (68.2)	45 (58.4)	0.28
PNI at listing (IQR)	41.65 (39.03, 42.66)	43.71 (43.39, 43.97)	47.62 (46.75, 50.31)	51.13 (47.83, 53.86)	<0.001
PNI before LT (IQR)	40.88 (38.95, 42.41)	47.66 (46.05, 49.02)	42.40 (40.22, 43.94)	50.47 (47.84, 51.91)	<0.001
Waiting period, months (IQR)	26.0 (11.0, 31.5)	25.0 (19.0, 26.0)	24.0 (13.5, 31.5)	27.0 (14.0, 34.0)	0.28
Primary disease, n (%)					0.26
IIPs	16 (50.0)	3 (33.3)	9 (40.9)	30 (39.0)	
CTD-ILD	8 (25.0)	2 (22.2)	2 (9.1)	5 (6.5)	
PH	1 (3.1)	2 (22.2)	3 (13.6)	16 (20.8)	
Bronchiectasis	3 (9.4)	0 (0.0)	2 (9.1)	5 (6.5)	
GVHD	1 (3.1)	1 (11.1)	2 (9.1)	4 (5.2)	
COPD	0 (0.0)	1 (11.1)	1 (4.5)	5 (6.5)	
BO	0 (0.0)	0 (0.0)	0 (0.0)	4 (5.2)	
Others	3 (9.4)	0 (0.0)	3 (13.6)	8 (10.4)	

Abbreviations: IQR, interquartile range; LT, lung transplantation; BMI, body mass index; PNI, prognostic nutritional index; IIPs, idiopathic interstitial pneumonias; PH, pulmonary hypertension; CTD-ILD, connective tissue disease-interstitial lung disease; GVHD, graft-versus-host disease; COPD, chronic obstructive pulmonary disease; BO, bronchiolitis obliterans. “Before LT” indicates immediately before lung transplantation (within 24 h). Values are median (IQR) or n (%). PNI cutoffs: 45. P-values calculated using Mann-Whitney U test for continuous variables, and chi-square test or Fisher’s exact test for categorical variables, as appropriate

**Fig. 4:**
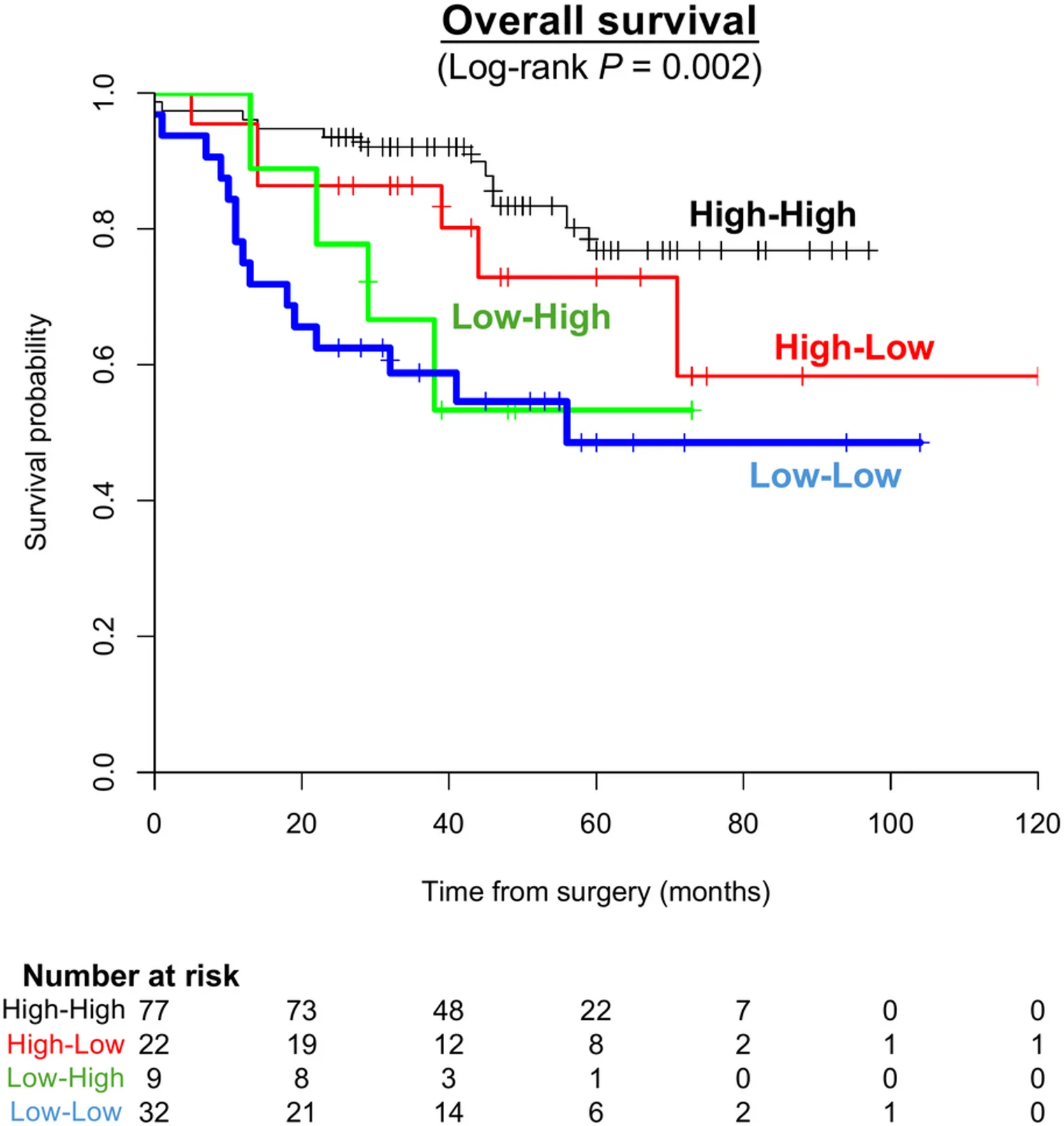
Overall survival curves stratified by the prognostic nutritional index trajectory groups

Kaplan–Meier survival curves showed significant overall differences among the four groups (log-rank *P* = 0.002) (Fig. 4). In pairwise comparisons using a Bonferroni-corrected significance threshold of 0.008 (0.05/6), the low-low group showed significantly worse survival than the high-high group (*P* < 0.001), with no significant differences between the other groups (Table 5). A multivariable analysis using a Cox model adjusted for age, sex, and primary disease was performed. The low-low group showed the poorest survival, and the high-high group had the most favorable survival (HR for low-low vs. high-high, 3.58; 95% CI, 1.62–7.91; *P* = 0.002). In contrast, an improvement in the PNI after listing (the low-high group) did not show a significantly better survival than the low-low group (HR, 1.15; 95% CI, 0.38–3.50; *P* = 0.81), with limited statistical power to detect this difference. Other comparisons were not statistically significant (Table 6). A visual inspection of the Kaplan–Meier curves for the four trajectory groups showed no marked crossing, and the Schoenfeld residual test confirmed that the proportional hazards assumption was satisfied for the trajectory Cox model (global *p* = 0.434).

**Table 5:** Pairwise comparisons of overall survival among PNI trajectory groups

	HR (95% CI)	*P* value
Low-Low vs. High-High	3.85 (1.75–8.33)	<0.001
Low-Low vs. High-Low	2.13 (0.83–5.56)	0.12
Low-Low vs. Low-High	1.19 (0.39–3.57)	0.76
Low-High vs. High-High	3.70 (1.18–11.11)	0.03
Low-High vs. High-Low	1.92 (0.53–6.67)	0.32
High-Low vs. High-High	1.75 (0.66–4.76)	0.26

Abbreviations: CI, confidence interval; HR, hazard ratio. Hazard ratios were calculated using univariable Cox proportional hazards regression. The second group in each comparison serves as the reference. Bonferroni-corrected significance threshold: *P* < 0.008 (0.05/6 comparisons)

**Table 6:** Multivariable Cox regression analysis for overall survival among PNI trajectory groups

	HR (95% CI)	*P* value
Low-Low vs. High-High	3.58 (1.62–7.91)	0.002
Low-Low vs. High-Low	1.67 (0.63–4.41)	0.30
Low-Low vs. Low-High	1.15 (0.38–3.50)	0.81
Low-High vs. High-High	3.11 (0.99–9.79)	0.05
Low-High vs. High-Low	1.45 (0.40–5.26)	0.57
High-Low vs. High-High	2.14 (0.79–5.80)	0.13

Abbreviations: CI, confidence interval; HR, hazard ratio. All models were adjusted for age, sex, and primary disease (interstitial lung disease vs. non-interstitial lung disease). The second group in each comparison serves as the reference. Bonferroni-corrected significance threshold: *P* < 0.008 (0.05/6 comparisons)

## Discussion

This study showed that the PNI calculated at listing or immediately before LT was a significant predictor of post-transplant outcomes. Furthermore, a trajectory analysis demonstrated that patients with a persistently high PNI had a significantly better survival than those with a persistently low PNI, while improvement in the PNI observed only after listing did not show a significant effect. These results suggest that the nutritional status at both time points, particularly at listing, is crucial and that earlier rather than later nutritional management may be more important for improving post-transplant outcomes, although this warrants confirmation in larger studies.

Similar to the present study, previous studies have also shown an association between PNI before LT and post-transplant outcomes [9, 13]. However, a distinctive feature of our study is the specification of two time points of PNI measurement (at listing and immediately before LT), which have not been clearly addressed in previous studies. This approach enabled us to evaluate the prognostic value of the PNI at each time point and analyze the trajectory changes in nutritional status. The larger separation of the Kaplan–Meier curves and a higher HR at listing than immediately before LT suggest that the PNI at listing more effectively identifies patients with a poor prognosis than the PNI immediately before LT.

Importantly, we did not find a significant improvement in survival even if the PNI was improved only after listing (low-low vs. low-high), suggesting that earlier rather than later nutritional optimization may be more beneficial, though a causal relationship remains to be established. This idea of early nutritional intervention is reasonable. Indeed, in patients with cirrhosis, who are potential candidates for liver transplantation, early assessment of the nutritional status and frailty, with intervention as required, is recommended [19]. Furthermore, nutritional intervention was reported to reduce post-transplant bacteremia in liver transplantation [20]. Nevertheless, nutritional changes after listing may still affect post-transplant outcomes. Unlike the large survival difference between the persistently high PNI group and persistently low PNI group, the difference in survival between the low-high group and high-high group did not reach significance (Table 6). The absence of a significant difference between the low-high and low-low groups should not be interpreted as evidence that nutritional improvement after listing is ineffective, as this may reflect limited statistical power given the small sample size (*n* = 9). Examination of the effect of nutritional changes after listing in a larger cohort and exploration of effective nutritional intervention for LT candidates after listing are important future research directions.

Taken together, the findings regarding the PNI at each time point and the trajectory changes have important clinical implications. The PNI at listing may serve primarily as a tool for baseline risk stratification and for identifying patients who may benefit from early nutritional support, whereas the PNI immediately before LT may provide a contemporaneous operative risk assessment and inform perioperative management decisions. The superior prognostic value of the PNI at listing underscores the importance of nutritional assessment from the time point before listing for patients expected to require LT, and the complementary use of both measurements may enable a more dynamic approach to nutritional assessment throughout the waiting period.

Our study has several limitations. First, this single-center, retrospective analysis was restricted to patients who underwent LT, and patients who died on the waiting list were not included. As previously reported from our institution, approximately 30% of the patients registered for lung transplantation from donation after brain death died while awaiting transplantation [21]. While the present analysis appropriately addresses the prognostic value of PNI in patients who reached transplantation, a complementary investigation focusing on waitlist outcomes among all listed candidates would be valuable to fully characterize the clinical impact of nutritional status across the entire transplant pathway. Additionally, the relatively small sample size limited statistical power for subgroup analyses, particularly for PNI trajectory comparisons. Second, no formal nutritional intervention pathway was implemented during the study period. While this means that we cannot directly inform on the efficacy of structured nutritional intervention, it also implies that the observed association between PNI and post-transplant outcomes was not confounded by intervention effects, providing a clean estimate of the natural prognostic relationship. Third, we focused on the long-term survival without analyzing other important outcomes, such as chronic lung allograft dysfunction or quality of life measures. Multicenter collaborative studies with larger cohorts would provide more robust evidence for the benefit of nutritional interventions and validate optimal PNI thresholds. Fourth, the findings may not be directly generalizable to transplant programs with shorter waiting periods or to those with a high proportion of living-donor lobar lung transplantation, in which the clinical trajectory of nutritional status before transplantation may differ substantially. Fifth, dichotomization of PNI at a cutoff of 45, while clinically pragmatic, may result in a loss of statistical information. However, sensitivity analyses treating the PNI as a continuous variable yielded consistent results.

Future research should focus on several directions. First, serial PNI measurements throughout the waiting period could help identify optimal monitoring intervals and critical time points for intervention. This approach may enhance risk stratification and inform decision-making regarding the timing of LT, balancing the risks of prolonged waiting against the potential benefits of nutritional optimization. Second, integration of the PNI with other frailty metrics might improve risk assessment. Additionally, examining the mechanisms linking PNI to the outcomes—whether through enhanced wound healing, improved immunity, or better tolerance of immunosuppression—would inform targeted interventions. Third, the nutritional status is intrinsically a modifiable variable, and our findings provide a rationale for considering it as a potential intervention target. Whether targeted nutritional intervention can translate into improved post-transplant outcomes warrants investigation in prospective interventional studies. We are currently conducting an exploratory trial of anamorelin—a ghrelin-receptor agonist approved for cancer cachexia— in patients with idiopathic pleuroparenchymal fibroelastosis awaiting LT (jRCTs031250402).

In conclusion, the PNI measured at listing and immediately before LT provides useful prognostic discrimination, and their complementary use enhances clinical efficacy. Patients with a low PNI at listing have a significantly increased risk of mortality. These findings underscore the importance of monitoring the nutritional status before transplant listing, highlighting the potential benefits of early nutritional intervention. Larger studies may clarify whether nutritional trajectory changes after listing influence the post-transplant prognosis.

## Supplementary Information

Below is the link to the electronic supplementary material.

Supplementary Material 1

Supplementary Material 2

Supplementary Material 3 Receiver operating characteristic curves for the prognostic nutritional index at listing (a) and immediately before lung transplantation (b) for overall mortality
