# Supplementary material for: Clinical impact of the prognostic nutritional index at listing and immediately before surgery in lung transplantation
Source: Surg Today. 2026 Jul 24;56(11):2846–55. doi: 10.1007/s00595-026-03421-x (PMC13722770; doi:10.1007/s00595-026-03421-x)
Supplement: Supplementary file 1 [file 595_2026_3421_MOESM1_ESM.docx]

**Supplementary Table 1.** Sensitivity analysis: hazard ratios of low PNI for overall survival across different adjustment sets

|  | PNI at listing | | |  | PNI before LT | | |
| --- | --- | --- | --- | --- | --- | --- | --- |
|  | HR | 95% CI | *P* value |  | HR | 95% CI | *P* value |
| Model 1 | 3.02 | 1.54–5.94 | 0.001 |  | 2.56 | 1.32–4.98 | 0.006 |
| Model 2 | 2.82 | 1.44–5.53 | 0.003 |  | 2.44 | 1.25–4.77 | 0.009 |
| Model 3 | 2.85 | 1.45–5.59 | 0.002 |  | 2.34 | 1.19–4.63 | 0.01 |

Abbreviations: HR, hazard ratio; CI, confidence interval; LT, lung transplantation; PNI, prognostic nutritional index; ILD, interstitial lung disease. "Before LT" indicates immediately before lung transplantation (within 24 hours). Primary disease was categorized as ILD (comprising idiopathic interstitial pneumonias and connective tissue disease-associated interstitial lung disease) versus non-ILD. Transplant type indicates bilateral versus single lung transplantation. Total number of death events: 37. Model 1 was adjusted for age and sex; Model 2 was adjusted for age, sex, and primary disease; Model 3 was adjusted for age, sex, primary disease, and transplant type.
