# Supplementary material for: Clinical impact of the prognostic nutritional index at listing and immediately before surgery in lung transplantation
Source: Surg Today. 2026 Jul 24;56(11):2846–55. doi: 10.1007/s00595-026-03421-x (PMC13722770; doi:10.1007/s00595-026-03421-x)
Supplement: Supplementary file 2 [file 595_2026_3421_MOESM2_ESM.docx]

**Supplementary Table 2.** Sensitivity analysis of multivariable Cox regression for overall survival treating PNI as a continuous variable

|  | PNI at listing | | |  | PNI before LT | | |
| --- | --- | --- | --- | --- | --- | --- | --- |
|  | HR | 95% CI | *P* value |  | HR | 95% CI | *P* value |
| Age, years | 0.99 | 0.96–1.01 | 0.28 |  | 0.98 | 0.96–1.01 | 0.24 |
| PNI (per 1-unit decrease) | 1.06 | 1.01–1.12 | 0.04 |  | 1.08 | 1.02–1.15 | 0.01 |
| Male | 2.64 | 1.23–5.67 | 0.01 |  | 2.49 | 1.16–5.37 | 0.02 |
| ILD | 2.48 | 1.14–5.39 | 0.02 |  | 2.46 | 1.12–5.41 | 0.03 |

Abbreviations: HR, hazard ratio; CI, confidence interval; LT, lung transplantation; PNI, prognostic nutritional index; ILD, interstitial lung disease. "Before LT" indicates immediately before lung transplantation (within 24 hours). Primary disease was categorized as ILD (comprising idiopathic interstitial pneumonias and connective tissue disease-interstitial lung disease) versus non-ILD. **The multivariable Cox regression model was adjusted for age, sex, and primary disease (ILD vs non-ILD), with PNI modeled as a continuous variable (per 1-unit decrease).** Total number of death events: 37.
